# The Influence of the Adsorbents Used on Changes in the Quality Parameters of Pumpkin Seed Oil as a Result of a Single-Stage Refining Process

**DOI:** 10.3390/molecules31071155

**Published:** 2026-03-31

**Authors:** Kamil Czwartkowski, Edyta Nizio, Damian Marcinkowski, Dominik Kmiecik, Anna Grygier, Aleksander Siger, Wojciech Golimowski

**Affiliations:** 1 Department of Agroengineering and Quality Analysis, Faculty of Production Engineering, Wroclaw University of Economics and Business, Komandorska 118/120, 53-345 Wroclaw, Poland; edyta.nizio@ue.wroc.pl (E.N.); wojciech.golimowski@ue.wroc.pl (W.G.); 2 Department of Inorganic Chemistry, Faculty of Production Engineering, Wroclaw University of Economics and Business, Komandorska 118/120, 53-345 Wroclaw, Poland; damian.marcinkowski@ue.wroc.pl; 3 Department of Food Technology of Plant Origin, Faculty of Food Science and Nutrition, Poznan University of Life Sciences, Wojska Polskiego 31, 60-634 Poznan, Poland; dominik.kmiecik@up.poznan.pl (D.K.); anna.grygier@up.poznan.pl (A.G.); 4 Department of Food Biochemistry and Analysis, Faculty of Food Science and Nutrition, Poznan University of Life Sciences, Wojska Polskiego 31, 60-634 Poznan, Poland; aleksander.siger@up.poznan.pl

**Keywords:** pumpkin seed oil, bleaching process, fatty acid profile, sterol content, tocopherol content

## Abstract

This study aimed to evaluate the impact of low- and high-temperature bleaching processes on the quality parameters of pumpkin seed oil. The research focused on optimizing the process to improve the oil’s physicochemical properties while reducing losses of valuable bioactive components. The bleaching process was carried out using 12 adsorbents in four technological variants, differing in temperature and adsorbent amount (30 °C/2% *w*/*w*, 30 °C/5%, 90 °C/2%, and 90 °C/5%). The scope of the analyses included, among others, the determination of acid (AV), peroxide (POV), and anisidine values (AnV), as well as the characterization of the fatty acid profile and the content of phytosterols and tocopherols. The data obtained were subjected to principal component analysis (PCA) to correlate the type of adsorbent with the process effects. It was shown that bleaching partially improves the oil’s quality parameters, though it is associated with a reduction in tocopherol and carotenoid content. Aluminum oxides are very poor adsorbents of vegetable oil components. Finely divided activated carbons exhibit the broadest spectrum of adsorbed components. Furthermore, bleaching earths have different effects on oil components depending on their composition and process temperature.

## 1. Introduction

Vegetable oils produced on a large scale, such as rapeseed oil and sunflower oil, undergo a refining process, the primary purpose of which is to remove non-fat substances that may adversely affect the shelf life, oxidative stability, and sensory properties of the final product [1]. This process enables the production of oil with consistent quality parameters, extended shelf life, and a wide range of applications in the food industry [2]. Refining processes lead to a significant reduction in the biological value of oil, resulting from the elimination of numerous bioactive compounds, such as natural antioxidants, vitamin precursors, phytosterols, and phenolic compounds [3].

The technology of vegetable oil refining comprises four basic stages: degumming, deacidification, bleaching, and deodorization. As a result of these processes, the oil is stripped of free fatty acids, phospholipids, mucilage, pigments, and trace metals [4]. In the final stage, volatile substances responsible for the intense smell and taste are removed [5].

Of the refining processes listed, bleaching is considered the least invasive to triacylglycerol structure and the least burdensome in terms of technology [6]. It does not require advanced equipment or extreme process conditions [7]. For this reason, bleaching is the most promising stage of refining that can be implemented in small-scale production or artisanal processing [8]. This process involves contacting the oil with a suitable adsorbent, such as bleaching earth, activated carbon, or a mixture thereof, resulting in the physical adsorption of selected components onto the adsorbent surface [9]. After the process is complete, the adsorbent material is mechanically separated from the oil [10]. The effectiveness of bleaching depends on many factors, including the type, quality, and dose of the adsorbent, contact time, process temperature, and the chemical properties of the oil itself. The literature shows that using different adsorbents can lead to significant differences in the profiles and degrees of removal of individual compounds [11].

Pumpkin oil is most often obtained by pressing pumpkin seeds belonging to the *Cucurbita* spp. *genus*. The Cucurbitaceae family includes about 27 plant species, but the agricultural and processing industries mainly use *Cucurbita pepo*, *Cucurbita maxima*, and *Cucurbita moschata* [12]. The seeds from which the oil is obtained usually constitute up to 5% *w*/*w* of the pumpkin and contain up to 40% of oil [13]. Pumpkin oil is characterized by high nutritional value and a unique chemical composition, which translates into its attributed health-promoting properties [14]. The literature on the subject indicates its potential supportive role in the prevention of cardiovascular diseases and disorders of the urinary tract and the prostate gland [15]. These properties are primarily due to the presence of numerous bioactive compounds, including carotenoids, tocopherols, plant sterols, and other antioxidants [16].

In practice, a commonly used method to increase the efficiency of pumpkin seed pressing is to roast the seeds at a temperature above 100 °C beforehand [17]. As a result of exposure to high temperatures, a series of Maillard reactions occurs in the raw material, giving the oil a characteristic dark, intense color and a specific sensory profile. This process leads to an increase in the content of free fatty acids (FFA), lipid degradation products, and other substances that can negatively affect the basic quality parameters of the oil, including its oxidative stability and suitability for more extended storage [18,19].

In view of the above, this study aims to evaluate the feasibility of using the pumpkin oil bleaching process to improve its quality parameters while minimizing loss of valuable bioactive components. Particular emphasis was placed on analyzing the impact of adsorbent type and dose on the effectiveness of removing undesirable components, while preserving the compounds responsible for pumpkin oil’s health-promoting properties.

## 2. Results and Discussion

The quality parameters of the analyzed samples are summarized in Table A1, which is attached to this paper. The article discusses the influence of adsorbents on the quality parameters of pumpkin oil (Figure 1, Figure 2, Figure 3, Figure 4, Figure 5, Figure 6, Figure 7, Figure 8, Figure 9, Figure 10, Figure 11 and Figure 12).

Five dominant phytosterols were identified in phytosterols content (PSC): β-sitosterol (PS1), spinasterol (PS2), Δ7,22,25-stigmastatrienol (PS3), Δ7-stigmastenol and Δ7,25-sigmastadienol (PS4), and Δ7-avenasterol (PS5), as well as trace amounts of other phytosterols (PS6) and squalene (Sq).

In each of the oils analyzed, there was a reduction in free fatty acid content (decrease in AV), indicating improved fat quality and consistent with the research by Gharby et al. (2021) [20]. The high POV for crude oil reported by Neđeral et al. (2012) results from prior seed roasting [21]. However, this is accompanied by a decrease in carotenoid, squalene, and vitamin E content, indicating that the improvement in fat quality is associated with reduced bioactive component levels, particularly tocopherols. Van Hoed et al. (2017) showed that pumpkin oils obtained from previously roasted seeds contain significantly more tocopherols than those obtained from unroasted seeds [22]. Research by Almeida et al. (2019) shows that it is not possible to perform bleaching without reducing carotenoid content [23]. However, their adsorption can be limited by selecting the appropriate adsorbent and adjusting the adsorption conditions. A statistically significant change in the fatty acid profile (FAP) was also observed in the samples analyzed, especially in those bleached at 90 °C. The modification of FAP during bleaching was described using the example of different hemp oil varieties in Golimowski et al. (2023) [8]. In addition, it was shown that a statistically significant difference in PSC occurs for most adsorbents only in the 5%/90 °C variant. Kwaśnica et al. (2022) reported a significant change in the phytosterol profile during bleaching at 60 °C, suggesting that bleaching at lower temperatures will not affect phytosterol reduction [24]. Another interesting observation is that squalene is more effectively removed from oils at low temperatures. Its adsorption during bleaching is low (about 25%), as confirmed by the study of Nergiz & Celikkale (2010), which found that the bleaching stage is the least squalene-reducing of the refining stages [25]. It has been observed that some adsorbents, at a 2% dose, at 90 °C, increase POV. It may mean that the oil for these adsorbents oxidizes faster than pro-oxidants are adsorbed, or that bleaching material releases pro-oxidants.

The analysis of the main components of the compared physicochemical parameters effectively demonstrated the differences between oils after the adsorption process and virgin oil. The first two principal components for each analyzed adsorbent account for over 60% of the total variance, indicating reduced dimensionality and improved component separation. The dimensionality reduction achieved by principal component analysis (PCA) was assessed by comparing the number of original variables describing the studied system with the number of extracted principal components that accounted for the largest proportion of the total variance. PC1 serves as the main compositional gradient, reflecting the inverse relationship among the analyzed quality indicators (especially the fatty acid profile, sterol profile, and tocopherol content). PC2, on the other hand, considers changes in the content of natural pigments and fatty numbers (AV, POV, AnV).

**Figure 1:**
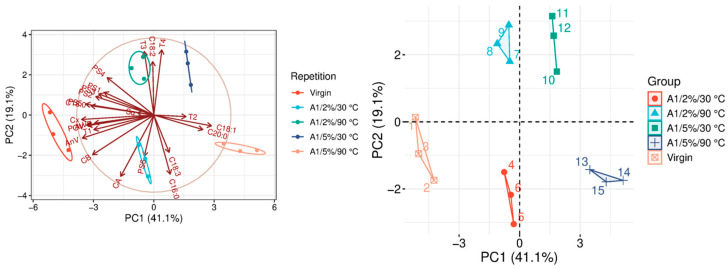
Principal component analysis of the influence of adsorbent A1 on virgin oil.

For A1 (Figure 1), both the amount of adsorbent added and the temperature at which the adsorption process is carried out significantly influence the adsorption efficiency. Variations in these parameters affect not only the removal of undesirable compounds but also the retention of valuable bioactive components naturally present in the oil. In all analyzed variants, a noticeable reduction in α-tocopherol was observed. Depending on the applied adsorption conditions, its content decreased by approximately 60–70%. It indicates a relatively strong affinity of the A1 adsorbent for this compound, which may negatively affect the oil’s antioxidant potential. When adsorption was performed at higher temperatures with 5% *w*/*w* adsorbent, A1 showed moderate effectiveness in improving selected quality parameters. Under these conditions, reductions in AV, POV, and AnV were observed, typically ranging from 10% to 20% of their initial values (Table A1). It suggests that A1 can partially remove both primary and secondary oxidation products. At elevated temperatures, A1 also removes carotenoids more effectively than chlorophylls. From a technological perspective, this may be considered unfavorable, as carotenoids contribute to the nutritional value and antioxidant properties of oils, whereas chlorophylls are generally less desirable. Importantly, adsorption with A1 does not significantly alter the oil’s phytosterol profile, indicating a low affinity of the adsorbent for these compounds.

**Figure 2:**
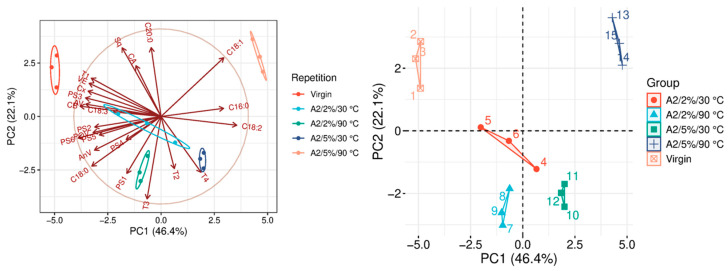
Principal component analysis of the influence of adsorbent A2 on virgin oil.

Adsorption using A2 (Figure 2) results in substantial changes in the composition of the analyzed oil and significantly affects several quality parameters. One of the most noticeable effects is the significant reduction in α-tocopherol content. Depending on the applied process conditions, the adsorption process may reduce the vitamin E level by up to seven times compared with the initial value. It indicates that A2 exhibits a high affinity for tocopherols, potentially significantly reducing the oil’s antioxidant potential. In addition to changes in minor bioactive compounds, A2 also shows a statistically significant influence on the fatty acid profile. The most pronounced changes were observed for C18:1, particularly under the conditions of 90 °C and 5% *w*/*w* adsorbent addition. Although the overall fatty acid composition remains relatively stable, these results suggest that more intensive process conditions may slightly alter the relative proportions of selected fatty acids. Statistical analysis also revealed similarities between several adsorption variants. Comparable results were observed for the conditions of 2% *w*/*w* at 30 °C, 2% *w*/*w* at 90 °C, and 5% *w*/*w* at 30 °C. These similarities indicate that the interaction between temperature and adsorbent dosage plays an important role in determining adsorption behavior. The results suggest that A2 becomes particularly active at higher doses and elevated temperatures. Under such conditions, the adsorbent demonstrates strong adsorption capacity, while at milder conditions it may act more selectively. However, it should be noted that in the 2%/90 °C variant, a significant increase in POV and AnV was observed, indicating a deterioration in oil quality and suggesting the possible promotion of oxidation processes.

**Figure 3:**
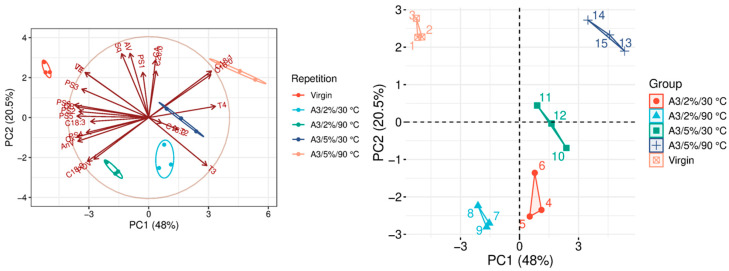
Principal component analysis of the influence of adsorbent A3 on virgin oil.

In FAP, no significant changes in the fatty acid ratio were observed after the A3 process (Figure 3). The oil after A3 in the 5%/90 °C variant differs statistically significantly from the other variants. It is mainly due to the removal of tocopherols, up to 80% for α-tocopherol and almost 50% for β-tocopherol (Table A1). At a 2% dose, it has no significant effect on carotenoid reduction. However, it shows a high capacity for chlorophyll removal, which may be important for the stability of these oils, as indicated by Suri et al. (2020) [26]. It is undoubtedly beneficial for improving the quality of vegetable oils. A3 also significantly reduces AnV, thereby decreasing fat decomposition products, mainly α- and β-unsaturated aldehydes, as suggested by the study by Wang et al. (2019) [27].

**Figure 4:**
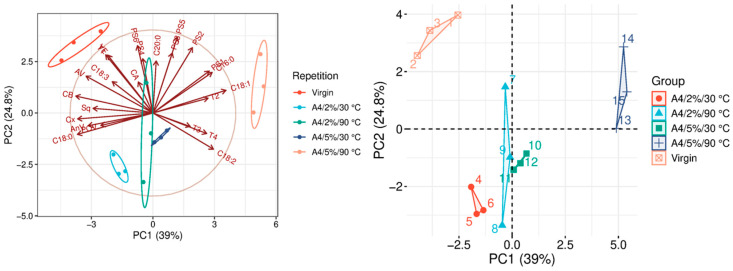
Principal component analysis of the influence of adsorbent A4 on virgin oil.

A4 applied at a dose of 2% demonstrated only slight effectiveness in the removal of chlorophylls and carotenoids, as illustrated in Figure 4. Despite the relatively limited pigment-removal capacity, the adsorbent showed a beneficial effect on other quality parameters of the oil. In particular, in the variant conducted at 30 °C, A4 contributed to a substantial reduction in the acid value (AV), lowering it by nearly 50%. It indicates that the material may partially improve oil stability and quality. At the same time, the adsorbent did not exhibit strong squalene-eliminating properties. It showed little activity toward phytosterols, suggesting that these valuable bioactive compounds were largely preserved during the treatment process. Similar to other adsorbents tested in the study, A4 caused a considerable decrease in α-tocopherol content, which may be considered a disadvantage given its antioxidant role in oils. Overall, given its influence on multiple physicochemical parameters, this adsorbent can be concluded to improve the oil’s overall quality characteristics. Furthermore, the statistical similarity observed among the three compared bleaching variants is noteworthy. Only the treatment at 5% adsorbent dose and 90 °C exhibited significantly higher adsorption capacity and a statistically significant effect on bioactive components, ultimately leading to noticeable changes in the fatty acid profile (FAP), as presented in Table A1.

**Figure 5:**
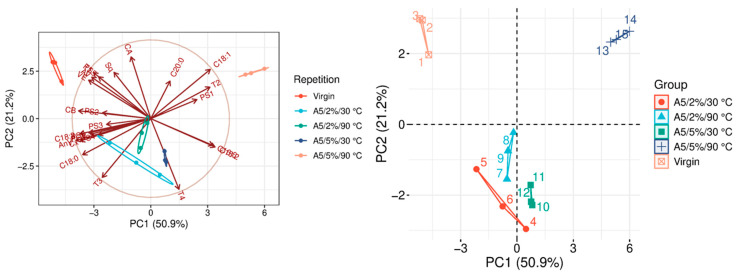
Principal component analysis of the influence of adsorbent A5 on virgin oil.

In the case of A5 (Figure 5), the factor that appears to initiate and most strongly influence oil quality improvement is the amount of the adsorbent added during the bleaching process. Variants using a 5% A5 dose show clearly better profiles for most bioactive components than those with lower dosages, with the notable exception of carotenoid content, which did not show a similar improvement. The parameter that most strongly differentiates the individual A5 bleaching variants is the tocopherol profile, particularly the concentrations of γ- and δ-tocopherol. In addition to tocopherols, significant differences between the variants are evident in oxidation-related parameters, such as anisidine value (AnV) and peroxide value (POV), indicating varying degrees of oxidative stability. When comparing A5-bleached oils with the raw oil, statistically significant differences were observed in the content of fatty acids C18:1 and C20:0. An exception to this pattern was the 5%/30 °C variant, which exhibited a fatty acid profile (FAP) similar to that of PSC and TC, as presented in Table A1.

**Figure 6:**
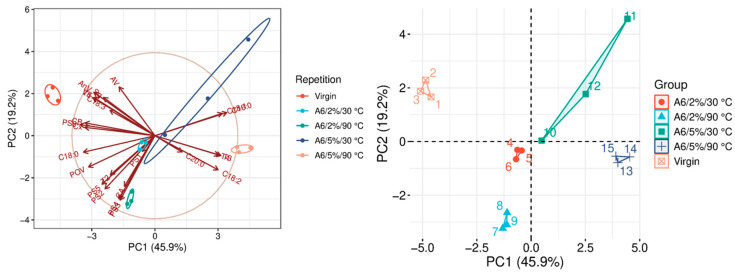
Principal component analysis of the influence of adsorbent A6 on virgin oil.

In the 5%/30 °C variant, A6 showed a statistically significant discrepancy between the results obtained (Figure 6). It was particularly evident in the removal of natural dyes and the small, statistically insignificant amount of squalene removed, which may be because A6 adsorbs other oil components much better. It is beneficial from the perspective of oil bioactivity. The slight effect of A6 on the content of γ- and δ-tocopherol and phytosterols is significant, which, according to the study by Ordoñez Lozada et al. (2021), is important from the point of view of the health-promoting properties of pumpkin oil [28]. The factor determining the differentiation of bleaching variants in the case of A6 is the change in FAP concerning C16:0 and C18:0 acids (Table A1). However, it should be noted that there is a very beneficial effect on the reduction in free fatty acids in all variants.

**Figure 7:**
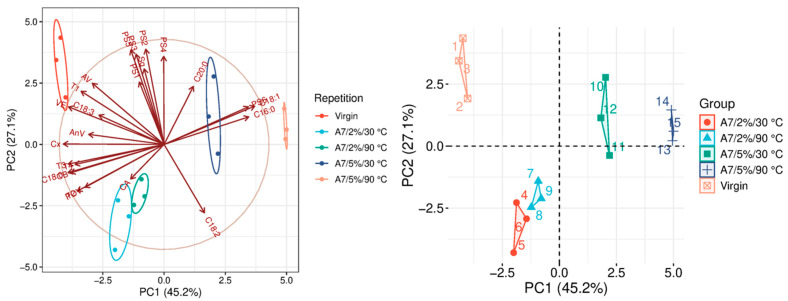
Principal component analysis of the influence of adsorbent A7 on virgin oil.

The parameter that most clearly differentiates the A7 variants (Figure 7) is the amount of bleaching earth applied during the process. In the variants using a 2% dose, A7 exhibits negligible adsorption capacity and therefore has a limited effect on improving the oil’s physicochemical properties. In contrast, when the dosage is increased to 5%, the adsorbent’s effectiveness becomes much more pronounced. In these variants, a particularly strong reduction in peroxide value (POV) is observed, suggesting improved oxidative stability of the oil. The 5% variants are also differentiated by changes in the fatty acid profile (FAP), including a significant decrease in C18:0 content exceeding 50%. Additionally, in the treatment conducted at 90 °C, an approximately 30% reduction in anisidine value (AnV) is observed. A slight decrease in carotenoids (about 10%) occurs in the 2% variants, accompanied by a simultaneous reduction in chlorophyll. However, in the 5% variants, A7 is among the most effective adsorbents for carotenoid removal (Table A1).

**Figure 8:**
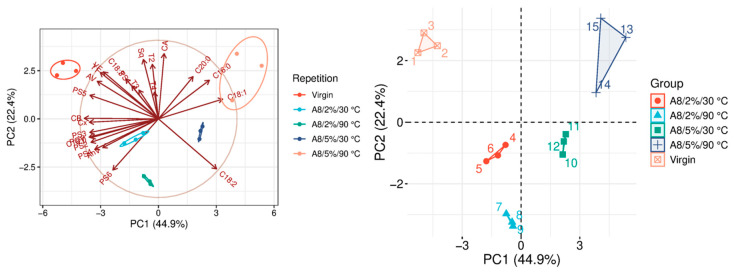
Principal component analysis of the influence of adsorbent A8 on virgin oil.

A1–A7 represent bleaching earths characterized by distinct physicochemical parameters that influence their adsorption efficiency and selectivity. Another group of adsorbents used in the study consists of activated carbons with different degrees of granulation, designated as A8–A10. Among them, A8 is the most finely ground activated carbon, characterized by a very high adsorption surface area of 1200–1500 m^2^/g. Owing to these properties, it demonstrates particularly strong adsorption capacity, especially at elevated temperatures (Figure 8). In the 5%/90 °C variant, A8 showed extremely intensive purification activity. Under these conditions, it almost completely removed free fatty acids, resulting in a very low acid value (AV = 0.20 mg KOH/g of oil). However, this strong adsorption capacity was also associated with the lowest vitamin E (VE) content among the tested variants, indicating a substantial removal of tocopherols during the process. Additionally, A8 significantly influenced the fatty acid profile (FAP), particularly affecting C18:0 and C18:1 levels. Despite these pronounced changes, its effect on squalene content in this configuration was statistically insignificant. In all 5% variants, the measured fatty acid quality indicators remained below 1.00, suggesting an improvement in overall oil stability. Nevertheless, this enhanced purification efficiency was accompanied by a considerable loss of pigments, resulting in a more than 60% reduction in carotenoid content, as shown in Table A1.

**Figure 9:**
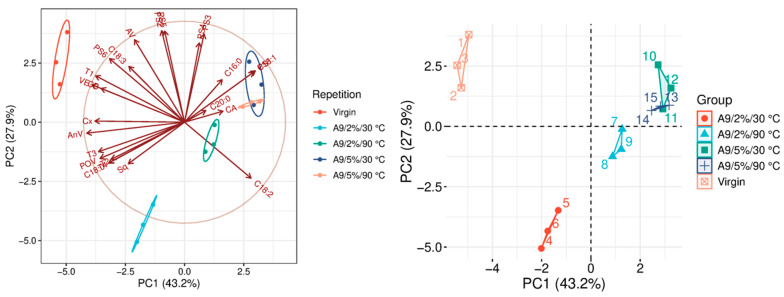
Principal component analysis of the influence of adsorbent A9 on virgin oil.

In turn, in A9 (medium-ground activated carbon with a specific surface area of 1000–1200 m^2^/g), the factor differentiating adsorption efficiency is the dose (Figure 9). In the variants, 5% is the most effective adsorbent. It has a significant effect on all analyzed factors except for squalene content. Furthermore, in these configurations, it significantly reduces tocopherol content (VE levels of 2.26 mg/100 g at 30 °C and 1.12 mg/100 g at 90 °C). None of the analyzed adsorbents is as effective as carotenoid removal (up to 80% effectiveness). In addition, the 90 °C variants significantly modify FAP (Table A1). This adsorbent is not recommended for improving oil parameters, but it can be used in refining processes, as indicated by Gharby (2022) [5].

**Figure 10:**
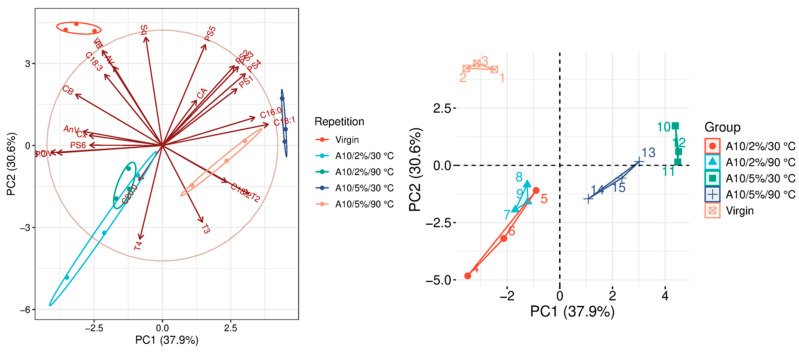
Principal component analysis of the influence of adsorbent A10 on virgin oil.

A10 is an activated carbon characterized by the smallest specific surface area among the tested carbons, ranging from 800 to 1000 m^2^/g. Due to its relatively lower surface area, it acts as a less aggressive adsorbent than other activated carbons (Figure 10). Despite its milder adsorption properties, A10 still improves several important quality parameters of the oil. It contributes to reducing acid value (AV), peroxide value (POV), and anisidine value (AnV), which are key indicators of hydrolytic and oxidative degradation. At the same time, this adsorbent does not significantly affect carotenoid content, suggesting that the natural pigments in the oil are largely preserved during the treatment. Another notable characteristic of A10 is the relatively high vitamin E (VE) content observed across all analyzed variants, ranging from 6.11 to 8.78 mg/100 g, indicating limited removal of tocopherols compared to stronger adsorbents. Furthermore, A10 does not significantly reduce squalene levels and does not influence phytosterol content (PSC). Statistically significant differences were observed only in the fatty acid profile (FAP), particularly for C18:0, which is consistent with trends seen for other activated carbons (Table A1).

**Figure 11:**
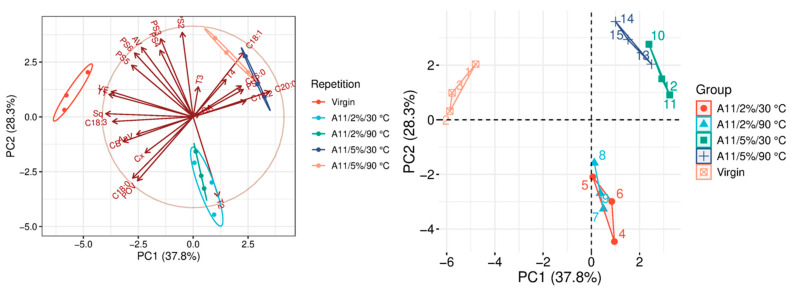
Principal component analysis of the influence of adsorbent A11 on virgin oil.

The last group of analyzed adsorbents consists of two types of aluminum oxide, designated as A11 and A12. In the case of A11 (Figure 11 and Table A1), the bleaching effectiveness is primarily determined by the amount of adsorbent added during treatment. At a 2% dose, A11 had only a minimal effect on most evaluated physicochemical parameters of the oil, indicating a relatively weak adsorption capacity under these conditions. Despite its limited impact on general quality indicators, the adsorbent caused noticeable, statistically significant changes in phytosterol (PSC) and total carotenoid (TC) levels. These alterations suggest that A11 may selectively interact with specific bioactive compounds rather than improving the oil’s overall quality. Based on the results, it can be concluded that A11 does not function effectively as an adsorbent to enhance oil quality parameters.

**Figure 12:**
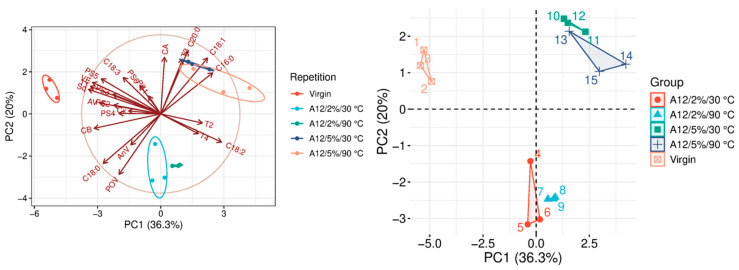
Principal component analysis of the influence of adsorbent A12 on virgin oil.

A12, similarly to A11, demonstrated greater effectiveness at higher doses (Figure 12). The activation of Al_2_O_3_ with acid resulted in a greater reduction in acid value (AV) than A11, indicating improved removal of free fatty acids. However, despite this advantage, A12 performed less favorably in terms of peroxide value (POV) and anisidine value (AnV), suggesting weaker control over oxidative degradation products. Additionally, A12 exerted a significantly stronger influence on phytosterol content (PSC) and total carotenoids (TC), resulting in greater changes in the composition of bioactive compounds in the oil (Table A1). Based on the results of both analyses, aluminum oxide adsorbents appear impractical for improving overall oil quality.

## 3. Materials and Methods

### 3.1. Description of the Bleaching Process

Twelve adsorbents (A1-A12) were used for bleaching, the characteristics of which are given in Table 1. The process was carried out in four variants (at 30 °C and 90 °C) with adsorbent concentrations of 2% and 5% *w*/*w*. 150 g ± 0.1 g of oil was weighed and heated to the process temperature, and then the specified dose of adsorbent was added. The adsorbent was mixed with the oil for 10 min on a magnetic stirrer at 1500 rpm, maintaining the specified process temperature. After this time, the oil was separated from the adsorbent on a qualitative paper filter under a vacuum of <100 mbar. The sample prepared in this way was centrifuged on a Thermo Scientific Legend X1R centrifuge (Thermo Fisher Scientific Inc., Waltham, MA, USA) at 20 °C and 15,000 rpm.

### 3.2. Acid Value (AV)

The acid number was determined in accordance with PN EN ISO 660:2020 and expressed in mg KOH/g of oil [29].

### 3.3. Peroxide Value (POV)

The peroxide value was determined in accordance with PN-EN ISO 3960:2017 and expressed as milliequivalents of active oxygen per kilogram of oil [meq O_2_/kg of oil] [30].

### 3.4. Anisidine Value (AnV)

The anisidine value was determined according to PN-EN ISO 6885:2008 [31].

### 3.5. Natural Dyes Content

The content of natural pigments was determined spectrophotometrically (distinguishing between chlorophyll A (*C_A_*), chlorophyll B (*C_B_*), and total carotenoid content (*C_x_*)) according to the methodology described by Alan Wellburn [32]. Approximately 1 g of oil (*m*) was placed in a glass test tube, and 20 mL of methanol (*D*) was added. The sample was then mixed using a vortex mini-shaker and stored in a dark room for 15 min to allow the phases to separate. 3 mL of the alcoholic phase was filtered through a 0.45 µm hydrophobic PTFE syringe filter and placed in a quartz spectrophotometric cuvette with an optical path length of 10 mm. The absorbance was measured at wavelengths of λ = 665.2 nm, λ = 652.4 nm, and λ = 470.0 nm on a SHIMADZU UV-1800 spectrophotometer (SHIMADZU Corporation, Kyoto, Japan). The results were expressed in mg/kg of oil.

(1) $$C_{A}=\frac{\left(16.72\;A_{665.2}-9.16\;A_{652.4}\right)\times D}{m}$$

(2) $$C_{B}=\frac{\left(34.09\;A_{652.4}-15.28\;A_{665.2}\right)\times D}{m}$$

(3) $$C_{x}=\frac{\left(\frac{1000\;A_{470.0}\;-\;1.63\;C_{a}\;-\;104.96\;C_{b}\;}{221}\right)\times D}{m}$$

### 3.6. Fatty Acid Profile (FAP)

The fatty acid profile was determined by gas chromatography after preparation of fatty acid methyl esters (FAME). The oils were saponified with 0.5 M KOH in methanol and then transesterified with BF_3_ solution in methanol according to the AOCS Ce 2–66 method [33]. The analysis was performed using an Agilent 7890A gas chromatograph equipped with a flame ionization detector (FID) (Agilent Technologies, Santa Clara, CA, USA) and an HP-88 capillary column (100 m × 0.25 mm × 0.20 μm) (Agilent Technologies, Santa Clara, CA, USA). Hydrogen was used as the carrier gas at a constant flow rate of 1.5 mL/min. The injector and detector temperatures were 250 °C and 280 °C, respectively. The column temperature program ranged from 120 to 230 °C. The FAME were identified by comparing with those of a commercial Supelco 37 Component FAME Mix (Merck KGaA, Darmstadt, Germany). All reagents used were GC or HPLC purity. The results are expressed as percentages of total fatty acids (%).

### 3.7. Phytosterols Content (PSC)

The phytosterol content was determined in three replicates using gas chromatography (GC-FID) with a Hewlett-Packard 6890 chromatograph (Hewlett-Packard, Palo Alto, CA, USA) equipped with a DB-35MS column (Agilent Technologies, Santa Clara, CA, USA). 50 µg of the internal standard (5α-cholestane) was added to 0.05 g of oil, and the samples were saponified with 1 M KOH in methanol. The unsaponifiable fraction was extracted with a hexane/methyl tert-butyl ether (1:1, *v*/*v*) mixture, evaporated under nitrogen, and silylated with BSTFA + 1% TMCS in anhydrous pyridine. The analysis was performed in splitless mode at injector and detector temperatures of 300 °C, with a furnace temperature program from 100 to 290 °C. Hydrogen (1.5 mL/min) was used as the carrier gas. Phytosterols were identified by their retention times relative to the standards [34]. All reagents used were of GC or HPLC purity and purchased from different suppliers: β-sitosterol no. 60-1016, Δ7-avenasterol no. 60-2017, and squalene no. 60-1017 from Larodan, Solna, Sweden; spinasterol PHL85776 from Merck, Rahway, NJ, USA; Δ7,22,25-stigmastatrienol no. HY-N5062 from MedChemExpress, Monmouth Junction, NJ, USA; Δ7-stigmastenol from TLC Standards, Newmarket, ON, Canada. The results were expressed in mg/g of oil.

### 3.8. Tocopherols Content (TC)

Tocochromanols were determined by HPLC after dissolving the oil samples in n-hexane. Analyses were performed using a Waters HPLC system (Waters Corporation, Milford, MA, USA) comprising a Waters 600 pump, a Waters 474 fluorescence detector, a Waters 2998 photodiode array detector (PDA), a Waters 2707 autosampler, and a Waters Jetstream 2 Plus column oven. Separation was carried out on a LiChrosorb Si 60 column (250 × 4.6 mm, 5 µm; Merck, Darmstadt, Germany) maintained at 20 °C. The mobile phase consisted of n-hexane/1,4-dioxane (96:4, *v*/*v*) delivered at a flow rate of 1.0 mL/min, and the injection volume was 10 µL. Fluorescence detection was performed at an excitation wavelength of 295 nm and emission wavelength of 330 nm, while the PDA detector was used to confirm the identity of the compounds based on their UV–Vis spectra. Identification of individual tocochromanols was performed by comparing their retention times with those of external standards. α-, β-, γ-, and δ-tocopherol standards (purity ≥95%, Merck, Darmstadt, Germany; catalog number 613424) were used for qualitative identification and quantitative calibration. The concentrations of individual compounds were calculated from calibration curves generated from standards and subsequently converted to vitamin E (*VE*) equivalents using the appropriate formula [35]. The parameters for validating the optimized method for the determination of tocopherols and tocotrienols were previously described by Siger et al. (2014) [35]. Vitamin E (*VE*) content was calculated using the following formula [36]:

(4) VE=1×T1+0.5×T2+0.1×T3+0.03×T4

where

*T*1—α-tocopherol,

*T*2—β-tocopherol,

*T*3—γ-tocopherol,

*T*4—δ-tocopherol.

The results are expressed in mg/100 g of oil for TC and mg/100 g for *VE*.

### 3.9. Statistical Analysis

Each analysis of the quality parameters of crude oil and oils after adsorption processes was performed three times. Results are presented as means and standard deviations. Principal component analysis (PCA) was performed for each adsorbent in different configurations relative to virgin oil using the SRplot platform developed by Tang et al. (2023) [37]. Before PCA analysis, Bartlett’s test was performed using Statistica 13.3 (StatSoft, Kraków, Poland). The Bartlett test demonstrated statistical significance of the correlation matrix (*p* < 0.05), confirming the existence of a relationship between the variables. Based on this, the data were deemed suitable for principal components analysis. The analyses show the distribution of oils after adsorption relative to virgin oil. On each resulting chart, the numbers 1–3 denote Virgin oil, 4–6 the 2%/30 °C adsorption variant, 7–9 the 2%/90 °C variant, 10–12 the 5%/30 °C variant, and 13–15 the 5%/90 °C variant.

## 4. Conclusions

The effects of 12 adsorbents across four different bleaching process configurations on the quality parameters of pumpkin seed oil were analyzed, including changes in tocopherol, phytosterol, fatty acid, carotenoid, and selected oxidation indicator content. This study demonstrates that the bleaching process can effectively improve the physicochemical quality of hot-pressed pumpkin seed oil by reducing undesirable pro-oxidants and pigments. While a partial reduction in natural antioxidants is inevitable during refining, the extent of this loss depends heavily on the chosen processing conditions. For practical applications, particularly in small-scale or artisanal oil production, it is highly recommended to conduct the bleaching process at a low temperature (30 °C) using a minimal adsorbent dose (2% *w*/*w*). Under these optimized parameters, using unmodified clays (such as magnesian bentonites) or selected finely ground activated carbons enables the selective removal of pigments while keeping degradation of valuable bioactive compounds, such as carotenoids and tocopherols, below 5%. Conversely, producers should strictly avoid high-temperature bleaching (e.g., 90 °C). Elevated temperatures not only drastically degrade heat-sensitive antioxidants but also accelerate the formation of primary and secondary oxidation products, which ultimately impair the oil’s oxidative stability. Implementing these low-temperature parameters provides the industry with a viable, technologically simple, and cost-effective method to enhance the shelf life and visual quality of pumpkin seed oil without compromising its inherent nutritional value. However, for some adsorbents (A2, A3, A4, and A10) used in the 2%/90 °C variant, increases in the peroxide value (PV) and anisidine value (AV) were observed, indicating an intensification of primary and secondary oxidation products. Carotenoids showed a similar trend, although in the case of finely ground carbon (A10) and some unmodified bleaching agents (A4, A5) used in the 2%/30 °C variant, their reduction did not exceed 5%. Activated carbons (A8 and A9), on the other hand, demonstrated the broadest adsorption spectrum for oil components. Therefore, they can only be used as an adjunct to precise adaptation processes. Aluminum oxides (A11 and A12) are ineffective adsorbents. However, it is necessary to analyze other parameters of the adsorbed oil, such as phosphorus or water content, as these adsorbents can be effective adsorbents.

## Figures and Tables

**Table 1:** Characteristics of the adsorbents used.

Code	Mineral	Modified	pH	Density [g∙dm^−3^]	Composition [%]						
					SiO_2_	Al_2_O_3_	Fe_2_O_3_		MgO	CaO	Na_2_O
A1	Attapulgite clay	Physically activated	8.0	410	55–60	2.5	12–14		18–21	0.5–1.0	0.05–0.25
A2	Attapulgite clay	pH-modified	3.2	510							
A3	Attapulgite clay	Not modified	8.0	470							
A4	Magnesian bentonite	Not modified	8.5	600	58.8	5.3	1.4		23.0	2.1	-
A5	Magnesian bentonite	Not modified	10.0	600	56.1	5.7	1.6		23.6	2.7	-
A6	Magnesian bentonite	Acid-modified	6.5	600	63.1	8.3	1.9		23.0	2.6	-
A7	Kerolite-hydrated magnesium silicate	Chemically modified	6.0	550	53.5	4.0	1.5		30.5	0.7	0.3
	Particle size [mm]	Surface Area [m^2^/g]									
A8	<0.1	1200–1500	>8.0	400–550	activated carbon min. 99%						
A9	<0.5	1000–1200	>8.0	250–300	activated carbon min. 99%						
A10	<1.5	800–1000	>8.0	180–210	activated carbon min. 99%						
	Variety										
A11	roasted, activated, neutral		7.0	390	Al_2_O_3_ min. 99%						
A12	roasted, activated, acidic		5.0	390	Al_2_O_3_ min. 99%						

## Data Availability

The original contributions presented in this study are included in the article. Further inquiries can be directed to the corresponding author.

## Abbreviations

The following abbreviations are used in this manuscript:

AV	acid value
POV	peroxide value
AnV	anisidine value
PCA	principal component analysis
FFA	free fatty acids
PSC	phytosterols content
PS1	β-sitosterol
PS2	spinasterol
PS3	Δ7,22,25-stigmastatrienol
PS4	Δ7-stigmastenol and Δ7,25-sigmastadienol
PS5	Δ7-avenasterol
PS6	other phytosterols
Sq	squalene
FAP	fatty acid profile
*C_A_*	chlorophyll A
*C_B_*	chlorophyll B
*C_x_*	carotenoids
FAME	fatty acid methyl esters
TC	tocopherols content
T1	α-tocopherol
T2	β-tocopherol
T3	γ-tocopherol
T4	δ-tocopherol
VE	vitamin E

## Appendix A

**Table A1:** Quality parameter values of the tested samples.

Sample		AV	POV	AnV	*C_A_*	*C_B_*	*C_x_*	FAP						Sq	PSC						TC				VE
								C16:0	C18:0	C18:1	C18:2	C18:3	C20:0		PS1	PS2	PS3	PS4	PS5	PS6	T1	T2	T3	T4	
Virgin oil		1.23 ± 0.02	5.42 ± 0.18	4.88 ± 0.15	7.70 ± 1.64	64.07 ± 7.06	25.61 ± 2.74	11.68 ± 0.04	5.90 ± 0.11	31.30 ± 0.11	50.49 ± 0.04	0.21 ± 0.00	0.42 ± 0.00	2.09 ± 0.11	0.14 ± 0.03	0.50 ± 0.02	0.56 ± 0.01	0.66 ± 0.01	0.44 ± 0.01	0.12 ± 0.00	10.32 ± 0.08	0.12 ± 0.00	45.29 ± 0.03	0.63 ± 0.01	14.93 ± 0.09
A1	2%/30 °C	1.22 ± 0.02	5.32 ± 0.11	3.73 ± 0.13	6.51 ± 1.09	52.22 ± 3.57	20.66 ± 0.49	11.77 ± 0.03	6.09 ± 0.00	30.93 ± 0.05	50.61 ± 0.01	0.19 ± 0.00	0.41 ± 0.00	1.54 ± 0.16	0.11 ± 0.01	0.42 ± 0.00	0.47 ± 0.02	0.55 ± 0.00	0.37 ± 0.01	0.12 ± 0.01	3.06 ± 0.04	0.12 ± 0.00	40.02 ± 0.02	0.59 ± 0.00	7.13 ± 0.04
	2%/90 °C	1.09 ± 0.01	4.96 ± 0.13	2.58 ± 0.28	3.31 ± 0.20	38.64 ± 0.78	15.53 ± 0.24	11.47 ± 0.13	5.63 ± 0.04	31.13 ± 0.06	51.23 ± 0.05	0.19 ± 0.00	0.36 ± 0.06	1.43 ± 0.02	0.12 ± 0.00	0.47 ± 0.01	0.53 ± 0.00	0.67 ± 0.01	0.38 ± 0.00	0.11 ± 0.03	3.17 ± 0.10	0.16 ± 0.02	45.43 ± 0.19	0.65 ± 0.01	7.81 ± 0.12
	5%/30 °C	0.63 ± 0.04	1.90 ± 0.04	0.96 ± 0.12	3.63 ± 0.25	37.27 ± 0.89	13.42 ± 1.06	11.44 ± 0.26	5.14 ± 0.09	31.92 ± 0.22	50.80 ± 0.00	0.18 ± 0.00	0.51 ± 0.05	2.01 ± 0.03	0.11 ± 0.01	0.44 ± 0.01	0.49 ± 0.00	0.59 ± 0.00	0.38 ± 0.01	0.10 ± 0.01	3.37 ± 0.09	0.11 ± 0.00	48.22 ± 0.08	0.72 ± 0.02	8.26 ± 0.09
	5%/90 °C	0.45 ± 0.03	1.15 ± 0.03	0.48 ± 0.09	5.41 ± 0.23	35.22 ± 0.77	4.04 ± 0.85	11.94 ± 0.05	3.44 ± 0.15	33.43 ± 0.12	50.44 ± 0.25	0.23 ± 0.03	0.54 ± 0.04	1.75 ± 0.02	0.10 ± 0.00	0.43 ± 0.02	0.49 ± 0.01	0.58 ± 0.03	0.35 ± 0.02	0.12 ± 0.00	3.27 ± 0.02	0.17 ± 0.00	42.25 ± 0.23	0.61 ± 0.01	7.60 ± 0.05
A2	2%/30 °C	1.12 ± 0.03	5.11 ± 0.02	4.28 ± 0.24	8.02 ± 2.39	47.52 ± 3.58	15.12 ± 3.69	11.62 ± 0.00	5.78 ± 0.08	31.35 ± 0.05	50.67 ± 0.04	0.18 ± 0.00	0.41 ± 0.00	1.53 ± 0.03	0.09 ± 0.04	0.36 ± 0.06	0.50 ± 0.01	0.59 ± 0.04	0.43 ± 0.00	0.11 ± 0.00	2.89 ± 0.05	0.12 ± 0.00	42.25 ± 0.16	0.62 ± 0.00	7.19 ± 0.03
	2%/90 °C	1.09 ± 0.02	6.19 ± 0.13	5.92 ± 0.62	2.99 ± 0.25	48.59 ± 0.36	8.15 ± 0.31	11.89 ± 0.15	5.93 ± 0.01	30.72 ± 0.04	50.91 ± 0.12	0.19 ± 0.00	0.37 ± 0.02	1.30 ± 0.06	0.13 ± 0.01	0.47 ± 0.01	0.52 ± 0.01	0.61 ± 0.02	0.35 ± 0.00	0.11 ± 0.00	2.29 ± 0.10	0.15 ± 0.00	41.65 ± 0.07	0.67 ± 0.02	6.55 ± 0.10
	5%/30 °C	0.79 ± 0.02	1.68 ± 0.07	2.78 ± 0.06	4.98 ± 0.81	35.85 ± 1.30	6.04 ± 0.84	11.90 ± 0.04	5.09 ± 0.04	31.57 ± 0.05	50.94 ± 0.05	0.20 ± 0.00	0.31 ± 0.00	1.97 ± 0.09	0.15 ± 0.04	0.49 ± 0.03	0.57 ± 0.03	0.64 ± 0.05	0.41 ± 0.02	0.11 ± 0.01	2.22 ± 0.02	0.13 ± 0.01	43.54 ± 0.01	0.72 ± 0.02	6.66 ± 0.03
	5%/90 °C	0.53 ± 0.04	1.95 ± 0.03	1.85 ± 0.18	6.15 ± 0.53	31.57 ± 1.32	4.77 ± 0.63	12.05 ± 0.01	2.05 ± 0.18	34.16 ± 0.19	51.12 ± 0.02	0.18 ± 0.01	0.44 ± 0.03	1.63 ± 0.08	0.13 ± 0.01	0.45 ± 0.02	0.52 ± 0.01	0.60 ± 0.02	0.35 ± 0.01	0.12 ± 0.00	0.20 ± 0.00	0.06 ± 0.01	21.62 ± 0.05	0.45 ± 0.03	2.40 ± 0.00
A3	2%/30 °C	1.14 ± 0.05	5.11 ± 0.06	3.95 ± 0.35	5.62 ± 2.93	47.96 ± 3.74	19.88 ± 1.03	11.73 ± 0.03	5.93 ± 0.05	31.12 ± 0.06	50.64 ± 0.02	0.18 ± 0.00	0.40 ± 0.00	1.83 ± 0.17	0.14 ± 0.05	0.50 ± 0.00	0.56 ± 0.03	0.69 ± 0.01	0.44 ± 0.01	0.09 ± 0.06	3.30 ± 0.01	0.11 ± 0.00	44.39 ± 0.14	0.63 ± 0.02	7.80 ± 0.03
	2%/90 °C	0.94 ± 0.02	7.83 ± 0.25	3.61 ± 0.32	4.20 ± 0.78	45.30 ± 4.52	18.47 ± 1.64	11.58 ± 0.13	5.80 ± 0.05	31.05 ± 0.14	51.09 ± 0.03	0.18 ± 0.00	0.29 ± 0.00	1.45 ± 0.04	0.16 ± 0.02	0.48 ± 0.04	0.55 ± 0.04	0.65 ± 0.03	0.41 ± 0.00	0.12 ± 0.01	3.06 ± 0.04	0.17 ± 0.01	46.57 ± 0.04	0.64 ± 0.02	7.82 ± 0.03
	5%/30 °C	0.56 ± 0.09	1.61 ± 0.09	2.15 ± 0.19	6.26 ± 0.28	43.26 ± 1.35	7.60 ± 0.39	11.91 ± 0.03	4.97 ± 0.09	32.72 ± 0.08	49.94 ± 0.07	0.21 ± 0.02	0.26 ± 0.06	2.07 ± 0.03	0.12 ± 0.00	0.47 ± 0.00	0.54 ± 0.01	0.65 ± 0.03	0.38 ± 0.02	0.10 ± 0.01	3.17 ± 0.03	0.12 ± 0.01	46.77 ± 0.02	0.66 ± 0.03	7.93 ± 0.03
	5%/90 °C	0.44 ± 0.05	2.16 ± 0.10	0.53 ± 0.18	7.93 ± 0.30	35.12 ± 0.63	1.47 ± 0.28	12.08 ± 0.02	1.78 ± 0.01	34.38 ± 0.04	51.15 ± 0.00	0.15 ± 0.01	0.46 ± 0.02	1.82 ± 0.05	0.12 ± 0.01	0.46 ± 0.02	0.52 ± 0.03	0.61 ± 0.03	0.37 ± 0.00	0.12 ± 0.01	1.60 ± 0.02	0.07 ± 0.01	34.65 ± 0.11	0.55 ± 0.02	5.12 ± 0.03
A4	2%/30 °C	0.77 ± 0.05	5.17 ± 0.04	5.48 ± 0.00	6.37 ± 0.35	43.92 ± 1.43	22.27 ± 0.17	11.54 ± 0.00	5.80 ± 0.10	31.28 ± 0.13	50.81 ± 0.04	0.18 ± 0.00	0.39 ± 0.00	1.60 ± 0.16	0.08 ± 0.00	0.41 ± 0.05	0.39 ± 0.13	0.61 ± 0.05	0.37 ± 0.00	0.13 ± 0.01	3.69 ± 0.09	0.12 ± 0.00	47.42 ± 0.17	0.64 ± 0.02	8.50 ± 0.11
	2%/90 °C	1.18 ± 0.02	8.63 ± 0.13	2.18 ± 0.08	3.96 ± 0.06	49.47 ± 0.75	22.24 ± 0.10	11.50 ± 0.21	5.92 ± 0.02	30.99 ± 0.11	51.11 ± 0.15	0.19 ± 0.00	0.30 ± 0.06	1.32 ± 0.03	0.10 ± 0.01	0.44 ± 0.02	0.50 ± 0.03	0.60 ± 0.05	0.35 ± 0.00	0.11 ± 0.01	3.42 ± 0.13	0.14 ± 0.00	48.44 ± 0.15	0.67 ± 0.01	8.35 ± 0.12
	5%/30 °C	0.64 ± 0.05	1.88 ± 0.11	4.12 ± 0.18	5.06 ± 0.23	45.15 ± 0.07	16.07 ± 0.25	11.85 ± 0.01	5.25 ± 0.08	31.63 ± 0.01	50.83 ± 0.07	0.11 ± 0.00	0.33 ± 0.02	1.84 ± 0.06	0.21 ± 0.02	0.52 ± 0.00	0.56 ± 0.03	0.62 ± 0.05	0.44 ± 0.01	0.11 ± 0.01	3.38 ± 0.10	0.14 ± 0.00	48.36 ± 0.09	0.73 ± 0.01	8.30 ± 0.11
	5%/90 °C	0.54 ± 0.02	0.81 ± 0.04	1.63 ± 0.23	6.67 ± 0.88	36.06 ± 1.50	7.95 ± 0.62	12.09 ± 0.01	1.66 ± 0.01	34.55 ± 0.04	51.13 ± 0.03	0.16 ± 0.01	0.42 ± 0.07	1.89 ± 0.01	0.11 ± 0.00	0.46 ± 0.01	0.52 ± 0.01	0.64 ± 0.02	0.38 ± 0.00	0.14 ± 0.00	1.59 ± 0.02	0.03 ± 0.00	15.30 ± 0.06	0.25 ± 0.02	3.14 ± 0.01
A5	2%/30 °C	0.87 ± 0.04	5.14 ± 0.29	4.05 ± 0.13	4.04 ± 0.42	51.72 ± 1.94	25.44 ± 0.99	11.98 ± 0.08	5.98 ± 0.00	30.67 ± 0.05	50.82 ± 0.00	0.19 ± 0.00	0.37 ± 0.03	1.23 ± 0.13	0.11 ± 0.02	0.37 ± 0.06	0.44 ± 0.06	0.56 ± 0.05	0.33 ± 0.04	0.13 ± 0.01	3.84 ± 0.01	0.14 ± 0.01	48.07 ± 0.04	0.67 ± 0.01	8.73 ± 0.00
	2%/90 °C	1.00 ± 0.01	6.25 ± 0.09	3.98 ± 0.20	5.46 ± 0.60	48.51 1.82	20.29 ± 0.44	11.83 ± 0.10	5.74 ± 0.01	30.85 ± 0.05	50.90 ± 0.00	0.19 ± 0.00	0.49 ± 0.04	1.61 ± 0.24	0.11 ± 0.02	0.43 ± 0.02	0.51 ± 0.04	0.61 ± 0.07	0.37 ± 0.04	0.11 ± 0.01	3.39 ± 0.11	0.16 ± 0.02	46.98 ± 0.11	0.66 ± 0.01	8.18 ± 0.09
	5%/30 °C	0.58 ± 0.04	2.30 ± 0.09	3.65 ± 0.14	5.11 ± 0.33	46.41 ± 0.83	18.17 ± 0.19	12.00 ± 0.10	4.87 ± 0.0	31.32 ± 0.00	51.24 ± 0.03	0.20 ± 0.00	0.39 ± 0.01	1.96 ± 0.12	0.16 ± 0.01	0.46 ± 0.03	0.54 ± 0.02	0.66 ± 0.02	0.40 ± 0.02	0.12 ± 0.00	3.30 ± 0.04	0.12 ± 0.00	47.60 ± 0.02	0.69 ± 0.02	8.14 ± 0.04
	5%/90 °C	0.47 ± 0.01	1.09 ± 0.15	1.26 ± 0.19	6.22 ± 0.36	35.80 ± 0.50	10.03 ± 0.13	12.07 ± 0.00	1.89 ± 0.12	34.29 ± 0.10	51.13 ± 0.01	0.16 ± 0.01	0.47 ± 0.01	1.97 ± 0.08	0.13 ± 0.01	0.49 ± 0.02	0.52 ± 0.01	0.67 ± 0.03	0.39 ± 0.02	0.13 ± 0.01	2.95 ± 0.03	0.09 ± 0.00	39.74 ± 0.13	0.59 ± 0.01	6.98 ± 0.02
A6	2%/30 °C	0.73 ± 0.00	5.38 ± 0.05	3.94 ± 0.32	6.21 ± 1.08	57.21 ± 1.78	22.98 ± 0.41	11.77 ± 0.01	5.93 ± 0.02	30.83 ± 0.01	51.00 ± 0.06	0.19 ± 0.00	0.30 ± 0.02	1.59 ± 0.01	0.13 ± 0.01	0.46 ± 0.01	0.53 ± 0.01	0.62 ± 0.02	0.38 ± 0.01	0.10 ± 0.00	3.83 ± 0.02	0.16 ± 0.00	48.42 ± 0.05	0.66 ± 0.00	8.77 ± 0.01
	2%/90 °C	0.70 ± 0.01	6.62 ± 0.06	3.48 ± 0.33	12.31 ± 1.02	49.08 ± 4.01	22.19 ± 1.38	11.74 ± 0.04	5.66 ± 0.02	31.06 ± 0.08	50.81 ± 0.05	0.18 ± 0.00	0.55 ± 0.06	1.51 ± 0.05	0.18 ± 0.01	0.47 ± 0.04	0.51 ± 0.01	0.72 ± 0.06	0.37 ± 0.02	0.11 ± 0.00	3.59 ± 0.05	0.13 ± 0.01	48.12 ± 0.07	0.66 ± 0.00	8.48 ± 0.05
	5%/30 °C	0.67 ± 0.01	2.38 ± 0.10	4.17 ± 0.19	5.39 ± 1.18	43.61 ± 3.59	18.86 ± 1.28	12.17 ± 0.01	2.59 ± 0.01	33.70 ± 0.04	50.81 ± 0.00	0.21 ± 0.03	0.53 ± 0.03	1.99 ± 0.00	0.12 ± 0.01	0.48 ± 0.01	0.55 ± 0.01	0.64 ± 0.01	0.40 ± 0.00	0.10 ± 0.00	3.06 ± 0.04	0.11 ± 0.00	45.54 ± 0.09	0.67 ± 0.01	7.69 ± 0.05
	5%/90 °C	0.54 ± 0.02	1.60 ± 0.06	2.50 ± 0.15	6.40 ± 0.26	39.76 ± 0.98	10.64 ± 0.89	12.09 ± 0.01	1.84 ± 0.19	34.33 ± 0.20	51.06 ± 0.02	0.17 ± 0.00	0.51 ± 0.04	1.58 ± 0.05	0.09 ± 0.01	0.45 ± 0.01	0.51 ± 0.00	0.64 ± 0.00	0.36 ± 0.01	0.13 ± 0.00	2.70 ± 0.06	0.07 ± 0.01	37.74 ± 0.18	0.56 ± 0.02	6.52 ± 0.08
A7	2%/30 °C	1.01 ± 0.02	5.35 ± 0.15	4.89 ± 0.16	6.09 ± 0.68	61.69 ± 7.45	22.17 ± 1.87	11.68 ± 0.08	5.86 ± 0.03	30.84 ± 0.06	51.16 ± 0.03	0.19 ± 0.00	0.27 ± 0.02	1.61 ± 0.02	0.17 ± 0.00	0.55 ± 0.01	0.62 ± 0.02	0.75 ± 0.03	0.43 ± 0.01	0.10 ± 0.01	3.87 ± 0.01	0.17 ± 0.00	48.50 ± 0.02	0.63 ± 0.00	8.82 ± 0.01
	2%/90 °C	0.73 ± 0.02	8.18 ± 0.13	1.69 ± 0.04	12.02 ± 0.15	53.06 ± 0.54	20.63 ± 0.41	11.82 ± 0.17	5.28 ± 0.04	31.51 ± 0.04	50.85 ± 0.06	0.18 ± 0.00	0.36 ± 0.04	1.58 ± 0.00	0.15 ± 0.01	0.44 ± 0.02	0.48 ± 0.00	0.62 ± 0.01	0.38 ± 0.01	0.10 ± 0.00	3.57 ± 0.04	0.15 ± 0.01	48.59 ± 0.02	0.68 ± 0.02	8.52 ± 0.04
	5%/30 °C	0.64 ± 0.03	1.01 ± 0.03	3.40 ± 0.20	5.59 ± 1.10	45.50 ± 2.22	16.95 ± 0.53	12.25 ± 0.00	2.20 ± 0.22	34.18 ± 0.20	50.49 ± 0.09	0.21 ± 0.01	0.67 ± 0.07	1.76 ± 0.02	0.14 ± 0.01	0.48 ± 0.02	0.56 ± 0.02	0.65 ± 0.01	0.39 ± 0.02	0.11 ± 0.00	3.73 ± 0.02	0.13 ± 0.01	48.34 ± 0.07	0.70 ± 0.01	8.65 ± 0.01
	5%/90 °C	0.60 ± 0.02	0.96 ± 0.06	1.35 ± 0.24	6.25 ± 0.00	33.70 ± 0.22	9.05 ± 0.19	12.12 ± 0.00	2.23 ± 0.06	33.92 ± 0.07	51.19 ± 0.00	0.16 ± 0.00	0.38 ± 0.01	1.64 ± 0.03	0.11 ± 0.01	0.45 ± 0.00	0.51 ± 0.01	0.62 ± 0.01	0.37 ± 0.01	0.13 ± 0.01	3.04 ± 0.04	0.09 ± 0.00	39.69 ± 0.12	0.60 ± 0.01	7.06 ± 0.05
A8	2%/30 °C	0.66 ± 0.04	5.32 ± 0.26	2.67 ± 0.05	6.37 ± 0.35	43.92 ± 1.43	22.27 ± 0.17	11.79 ± 0.12	5.86 ± 0.02	30.84 ± 0.05	50.87 ± 0.04	0.18 ± 0.00	0.46 ± 0.01	1.56 ± 0.04	0.13 ± 0.01	0.48 ± 0.05	0.55 ± 0.04	0.65 ± 0.05	0.40 ± 0.03	0.09 ± 0.01	3.88 ± 0.02	0.16 ± 0.01	46.42 ± 0.13	0.67 ± 0.01	8.62 ± 0.04
	2%/90 °C	0.51 ± 0.03	4.78 ± 0.09	6.36 ± 0.12	4.34 ± 0.62	47.48 ± 0.57	16.65 ± 1.38	11.35 ± 0.03	5.52 ± 0.02	31.51 ± 0.02	51.09 ± 0.02	0.18 ± 0.00	0.35 ± 0.02	1.80 ± 0.00	0.09 ± 0.01	0.41 ± 0.00	0.47 ± 0.00	0.62 ± 0.01	0.20 ± 0.14	0.08 ± 0.00	3.13 ± 0.01	0.12 ± 0.00	43.60 ± 0.06	0.65 ± 0.02	7.56 ± 0.01
	5%/30 °C	0.48 ± 0.01	0.97 ± 0.10	0.82 ± 0.05	6.03 ± 0.13	25.26 ± 0.48	18.66 ± 0.32	12.19 ± 0.01	1.75 ± 0.25	34.47 ± 0.25	51.03 ± 0.02	0.16 ± 0.02	0.40 ± 0.05	1.95 ± 0.00	0.19 ± 0.00	0.45 ± 0.00	0.53 ± 0.00	0.62 ± 0.02	0.40 ± 0.01	0.10 ± 0.00	3.32 ± 0.03	0.17 ± 0.01	38.72 ± 0.20	0.66 ± 0.00	7.29 ± 0.06
	5%/90 °C	0.20 ± 0.02	0.86 ± 0.10	0.80 ± 0.18	8.03 ± 0.33	21.81 ± 0.26	8.82 ± 0.20	12.20 ± 0.01	1.97 ± 0.08	34.13 ± 0.06	50.96 ± 0.03	0.19 ± 0.01	0.56 ± 0.05	2.08 ± 0.09	0.12 ± 0.02	0.35 ± 0.03	0.44 ± 0.03	0.56 ± 0.03	0.35 ± 0.02	0.07 ± 0.02	0.85 ± 0.04	0.01 ± 0.00	22.11 ± 0.02	0.42 ± 0.02	3.07 ± 0.04
A9	2%/30 °C	0.62 ± 0.04	5.17 ± 0.24	3.74 ± 0.12	8.75 ± 0.13	34.43 ± 1.31	18.79 ± 0.60	11.57 ± 0.01	5.84 ± 0.00	30.92 ± 0.03	51.04 ± 0.04	0.18 ± 0.00	0.45 ± 0.07	1.65 ± 0.07	0.15 ± 0.01	0.45 ± 0.02	0.48 ± 0.01	0.59 ± 0.04	0.39 ± 0.01	0.08 ± 0.00	2.47 ± 0.04	0.16 ± 0.01	39.26 ± 0.12	0.64 ± 0.01	6.49 ± 0.05
	2%/90 °C	0.38 ± 0.01	4.57 ± 0.12	1.32 ± 0.29	7.06 ± 0.16	38.75 ± 0.79	14.35 ± 0.31	11.17 ± 0.04	5.73 ± 0.04	31.30 ± 0.02	51.33 ± 0.01	0.18 ± 0.00	0.29 ± 0.01	1.82 ± 0.04	0.10 ± 0.02	0.44 ± 0.01	0.50 ± 0.00	0.59 ± 0.02	0.38 ± 0.00	0.09 ± 0.00	1.43 ± 0.08	0.09 ± 0.00	35.47 ± 0.18	0.59 ± 0.01	5.03 ± 0.06
	5%/30 °C	0.22 ± 0.02	1.02 ± 0.06	0.98 ± 0.17	10.86 ± 2.15	29.80 ± 7.29	12.55 ± 2.80	12.05 ± 0.00	2.09 ± 0.12	34.24 ± 0.13	50.96 ± 0.04	0.18 ± 0.01	0.48 ± 0.06	2.12 ± 0.04	0.16 ± 0.01	0.45 ± 0.00	0.52 ± 0.00	0.65 ± 0.01	0.39 ± 0.01	0.09 ± 0.00	0.29 ± 0.01	0.09 ± 0.01	19.11 ± 0.11	0.54 ± 0.01	2.26 ± 0.02
	5%/90 °C	0.18 ± 0.04	0.92 ± 0.05	0.26 ± 0.11	9.24 ± 0.23	23.92 ± 0.61	4.44 ± 0.23	12.17 ± 0.01	3.54 ± 0.03	32.59 ± 0.03	50.98 ± 0.03	0.19 ± 0.02	0.53 ± 0.01	2.18 ± 0.00	0.12 ± 0.01	0.42 ± 0.00	0.49 ± 0.00	0.60 ± 0.02	0.37 ± 0.02	0.08 ± 0.00	0.05 ± 0.00	0.06 ± 0.02	10.38 ± 0.12	0.33 ± 0.01	1.12 ± 0.01
A10	2%/30 °C	0.80 ± 0.02	5.15 ± 0.10	4.79 ± 0.16	7.11 ± 2.06	50.61 ± 4.28	25.37 ± 1.00	11.52 ± 0.03	5.90 ± 0.01	30.96 ± 0.04	50.98 ± 0.02	0.19 ± 0.00	0.46 ± 0.02	1.53 ± 0.01	0.17 ± 0.00	0.49 ± 0.00	0.51 ± 0.02	0.62 ± 0.05	0.38 ± 0.02	0.09 ± 0.00	3.75 ± 0.01	0.15 ± 0.00	49.38 ± 0.20	0.63 ± 0.01	8.78 ± 0.01
	2%/90 °C	0.95 ± 0.02	6.30 ± 0.09	4.62 ± 0.22	6.01 ± 0.83	51.61 ± 3.66	24.29 ± 1.51	11.29 ± 0.04	5.72 ± 0.07	30.58 ± 0.13	50.35 ± 0.03	0.19 ± 0.00	1.87 ± 0.06	1.65 ± 0.03	0.12 ± 0.02	0.48 ± 0.01	0.53 ± 0.01	0.69 ± 0.01	0.42 ± 0.00	0.10 ± 0.00	3.44 ± 0.03	0.12 ± 0.00	47.27 ± 0.05	0.65 ± 0.02	8.24 ± 0.02
	5%/30 °C	0.64 ± 0.02	1.12 ± 0.07	4.19 ± 0.15	10.41 ± 0.53	47.18 ± 0.71	22.23 ± 0.48	12.16 ± 0.01	1.85 ± 0.12	34.38 ± 0.10	50.96 ± 0.02	0.18 ± 0.00	0.46 ± 0.01	1.46 ± 0.07	0.19 ± 0.01	0.51 ± 0.02	0.62 ± 0.01	0.73 ± 0.01	0.43 ± 0.02	0.09 ± 0.01	3.40 ± 0.12	0.12 ± 0.00	46.49 ± 0.19	0.65 ± 0.01	8.12 ± 0.14
	5%/90 °C	0.60 ± 0.02	2.01 ± 0.05	3.35 ± 0.29	4.31 ± 0.31	49.45 ± 0.28	21.57 ± 0.72	12.22 ± 0.00	2.98 ± 0.06	33.15 ± 0.06	50.93 ± 0.06	0.20 ± 0.00	0.51 ± 0.05	1.97 ± 0.15	0.17 ± 0.01	0.46 ± 0.00	0.56 ± 0.01	0.66 ± 0.00	0.41 ± 0.00	0.08 ± 0.00	2.50 ± 0.01	0.08 ± 0.00	35.57 ± 0.13	0.54 ± 0.01	6.11 ± 0.00
A11	2%/30 °C	1.08 ± 0.02	5.13 ± 0.11	4.53 ± 0.07	8.57 ± 0.96	56.77 ± 3.07	25.07 ± 0.83	12.18 ± 0.09	5.92 ± 0.01	30.77 ± 0.07	50.50 ± 0.15	0.19 ± 0.00	0.44 ± 0.00	1.38 ± 0.03	0.15 ± 0.01	0.43 ± 0.02	0.51 ± 0.01	0.64 ± 0.00	0.40 ± 0.00	0.09 ± 0.01	3.34 ± 0.01	0.13 ± 0.00	46.55 ± 0.02	0.64 ± 0.02	8.07 ± 0.02
	2%/90 °C	1.02 ± 0.02	6.13 ± 0.06	4.30 ± 0.11	4.50 ± 0.33	57.74 ± 0.24	24.48 ± 0.84	11.41 ± 0.08	5.63 ± 0.01	31.27 ± 0.04	51.06 ± 0.02	0.19 ± 0.00	0.44 ± 0.01	1.81 ± 0.07	0.12 ± 0.03	0.44 ± 0.01	0.52 ± 0.01	0.61 ± 0.01	0.39 ± 0.00	0.08 ± 0.00	3.57 ± 0.04	0.12 ± 0.00	48.32 ± 0.05	0.68 ± 0.02	8.47 ± 0.04
	5%/30 °C	0.74 ± 0.10	1.28 ± 0.06	4.42 ± 0.40	11.38 ± 1.83	50.85 ± 0.25	21.79 ± 1.67	12.20 ± 0.00	2.20 ± 0.14	34.08 ± 0.14	50.87 ± 0.01	0.19 ± 0.00	0.48 ± 0.01	1.66 ± 0.01	0.19 ± 0.01	0.40 ± 0.07	0.48 ± 0.11	0.56 ± 0.16	0.30 ± 0.11	0.08 ± 0.02	3.34 ± 0.04	0.10 ± 0.01	47.14 ± 0.07	0.67 ± 0.00	8.12 ± 0.04
	5%/90 °C	0.64 ± 0.01	2.49 ± 0.11	4.14 ± 0.05	4.33 ± 0.51	56.08 ± 1.54	21.65 ± 0.21	12.18 ± 0.10	3.43 ± 0.02	32.72 ± 0.08	51.03 ± 0.02	0.18 ± 0.00	0.46 ± 0.02	1.92 ± 0.11	0.19 ± 0.00	0.46 ± 0.01	0.56 ± 0.01	0.56 ± 0.01	0.42 ± 0.01	0.09 ± 0.00	2.61 ± 0.05	0.07 ± 0.00	36.58 ± 0.29	0.54 ± 0.01	6.32 ± 0.08
A12	2%/30 °C	0.96 ± 0.03	5.10 ± 0.19	4.79 ± 0.10	1.21 ± 0.43	55.91 ± 0.59	24.85 ± 0.63	11.52 ± 0.21	5.80 ± 0.02	30.97 ± 0.05	51.14 ± 0.15	0.19 ± 0.00	0.39 ± 0.02	1.55 ± 0.01	0.16 ± 0.01	0.51 ± 0.01	0.57 ± 0.00	0.65 ± 0.05	0.42 ± 0.00	0.11 ± 0.00	3.16 ± 0.04	0.12 ± 0.01	46.21 ± 0.16	0.71 ± 0.05	7.85 ± 0.07
	2%/90 °C	0.83 ± 0.02	7.02 ± 0.16	4.90 ± 0.20	6.62 ± 0.15	48.47 ± 0.93	24.64 ± 0.79	12.07 ± 0.16	4.82 ± 0.14	31.67 ± 0.32	50.88 ± 0.03	0.19 ± 0.00	0.37 ± 0.01	1.89 ± 0.02	0.14 ± 0.01	0.43 ± 0.02	0.50 ± 0.02	0.58 ± 0.03	0.37 ± 0.03	0.08 ± 0.00	3.31 ± 0.05	0.13 ± 0.00	47.37 ± 0.12	0.72 ± 0.02	8.13 ± 0.06
	5%/30 °C	0.62 ± 0.05	1.67 ± 0.09	4.68 ± 0.22	10.75 ± 0.23	44.76 ± 1.54	24.42 ± 0.50	12.22 ± 0.01	2.29 ± 0.16	33.92 ± 0.19	50.94 ± 0.02	0.19 ± 0.01	0.44 ± 0.01	1.45 ± 0.02	0.11 ± 0.01	0.46 ± 0.01	0.55 ± 0.00	0.65 ± 0.01	0.38 ± 0.00	0.08 ± 0.00	3.62 ± 0.02	0.11 ± 0.01	48.35 ± 0.01	0.72 ± 0.01	8.53 ± 0.02
	5%/90 °C	0.57 ± 0.01	1.70 ± 0.15	4.53 ± 0.29	6.53 ± 0.08	47.04 ± 0.64	24.07 ± 0.74	12.30 ± 0.01	3.12 ± 0.10	32.87 ± 0.11	51.03 ± 0.00	0.20 ± 0.01	0.48 ± 0.02	1.99 ± 0.00	0.17 ± 0.01	0.47 ± 0.00	0.55 ± 0.01	0.65 ± 0.00	0.41 ± 0.01	0.08 ± 0.00	2.62 ± 0.02	0.08 ± 0.00	36.53 ± 0.09	0.55 ± 0.01	6.33 ± 0.02

AV—acid value [mg KOH/g of oil], POV—peroxide value [meq O_2_/kg of oil], AnV—anisidine value [-], *C_A_*—chlorophyll A [µg/g], *C_B_*—chlorophyll B [µg/g], *C_x_*—carotenoids [µg/g], FAP—fatty acids profile [%], Sq—squalene [mg/g], PSC—phytosterols content [mg/g], TC—tocopherols content [mg/100 g], VE—vitamin E content [mg/100 g].
